# *Plasmodium vivax* infection: a major determinant of severe anaemia in infancy

**DOI:** 10.1186/s12936-016-1373-8

**Published:** 2016-06-16

**Authors:** Enny Kenangalem, Muhammad Karyana, Lenny Burdarm, Shunmay Yeung, Julie A. Simpson, Emiliana Tjitra, Nicholas M. Anstey, Jeanne Rini Poespoprodjo, Ric N. Price, Nicholas M. Douglas

**Affiliations:** Timika Malaria Research Program, Papuan Health and Community Development Foundation, Timika, Papua Indonesia; Mimika District Health Authority, Timika, Papua Indonesia; National Institute of Health Research and Development, Ministry of Health, Jakarta, Indonesia; Faculty of Public Health and Policy, London School of Hygiene & Tropical Medicine, London, UK; Centre for Epidemiology and Biostatistics, Melbourne School of Population and Global Health, University of Melbourne, Melbourne, Australia; Global and Tropical Health Division, Menzies School of Health Research and Charles Darwin University, PO Box 41096, Casuarina, Darwin, 0811 Australia; Division of Medicine, Royal Darwin Hospital, Darwin, NT Australia; Department of Child Health, Faculty of Medicine, University Gadjah Mada, Yogyakarta, Indonesia; Centre for Tropical Medicine and Global Health, Nuffield Department of Clinical Medicine, University of Oxford, Oxford, UK; Division of Medicine, Christchurch Hospital, Christchurch, New Zealand

**Keywords:** Malaria, *Plasmodium falciparum*, *Plasmodium vivax*, *Plasmodium malariae*, Anaemia, Indonesia

## Abstract

**Background:**

Most malarious countries outside of Africa are co-endemic for *Plasmodium falciparum* and *Plasmodium vivax.* The comparative burden of anaemia in the community caused by these two species is incompletely characterized.

**Methods:**

A three-stage, cross-sectional, community survey was used to determine the proportion of moderate or severe anaemia (haemoglobin <7 g/dL) attributable to patent *P. vivax, P. falciparum* and mixed parasitaemia in Papua, Indonesia. Adjusted population-attributable fractions were calculated from multivariable logistic regression models. Eight hundred and twenty-five households were surveyed with a total of 5255 occupants, 3890 (74 %) of whom were present and provided a blood sample. *Plasmodium falciparum* parasitaemia was present in 8.1 % (n = 315) of participants, *P. vivax* in 6.4 % (n = 250) and mixed infections in 1.9 % (n = 72). Overall, *P. falciparum* was associated with a mean reduction in haemoglobin of 1.16 g/dL compared to those without patent parasitaemia [95 % confidence interval (95 % CI) 0.91, 1.41 g/dL]. The corresponding values for *P. vivax* and mixed infections were 0.66 g/dL (95 % CI 0.35, 0.96) and 1.25 g/dL (0.71, 1.80), respectively. Overall, 16.7 % (95 % CI 8.52, 24.2 %) of haemoglobin concentrations <7 g/dL in the community were estimated to be attributable to patent parasitaemia. The fractions for infants and 1–5 years old were 34.4 % (95 % CI −3.30, 58.3 %) and 23.2 % (95 % CI 3.34, 39.0 %), respectively. *Plasmodium vivax* was associated with a greater than threefold higher attributable fraction of anaemia in infants compared with *P. falciparum* [27.6 % (95 % CI −3.20, 49.2 %) versus 7.94 % (−5.87, 20.0 %)].

**Conclusion:**

Despite comparatively low-level endemicity, malaria is associated with a significant proportion of all cases of community anaemia in southern Papua. Contrary to its benign reputation, *P. vivax* is an important and preventable risk factor for anaemia during infancy—a probable consequence of relapsing disease prior to the development of immunity.

## Background

Anaemia is a common manifestation of *Plasmodium* infection [1, 2]. Its impact is most apparent in the hospital setting where it accounts for a substantial proportion of malaria morbidity and, to a lesser extent, mortality [3–6].

The burden of malarial anaemia outside of healthcare facilities is less well understood and its contribution to ‘indirect’ malaria morbidity and mortality is largely unknown [7]. There are two major explanations for this uncertainty. Firstly, the adverse effects of mild or moderate anaemia per se are not clearly understood. Haemoglobin concentrations below 7 g/dL probably confer an increased risk of poor pregnancy outcomes such as haemorrhagic shock [5], low birth weight [8, 9] and poor neurocognitive development [10, 11] but other, less tangible, effects such as decreased resilience to infectious diseases [11] remain unproven. Secondly, in hyper- and holo-endemic regions, the majority of individuals will be parasitized at any point in time, making it difficult to disentangle the impact of malaria from other concomitant causes of anaemia. To get around this problem, researchers in highly endemic settings have typically limited their assessments to individuals with clinical malaria, based on the presence of fever and a probabilistic cut-off for parasite density [12, 13]. This approach inevitably misses the haematological effects of low-density symptomatic infections and asymptomatic parasitaemia. Evidence for the likely importance of the latter comes from several sources, including intervention trials that have demonstrated much greater improvements in haemoglobin concentrations and mortality than could have been predicted by preventing symptomatic infections alone [14–16], as well as observational studies showing large variations in haemoglobin levels coinciding with seasonal fluctuations in parasite prevalence [17]. These and other findings led Molineaux to suggest that ‘total’ falciparum malaria mortality in Africa is likely to be twice as high as ‘direct’ malaria mortality [18]. Although there is relatively little information for *Plasmodium vivax,* one could assume that the ratio for this species would be even greater since direct deaths are rare (but under-estimated [4, 19–21]) and infections are associated with significant morbidity [1, 22–27]. The authors of the current study postulate that anaemia, caused by either *P. falciparum* or *P. vivax,* may underlie a substantial proportion of all ‘indirect’ malaria morbidity and mortality.

Southern Papua, like much of south Asia and Latin America, is co-endemic for *P. falciparum* and *P. vivax*. It has a lower entomological inoculation rate than most tropical African nations [one to four infective bites per year (unpublished data)] but a disproportionately high direct malaria-attributable mortality rate [19]. Such a setting provides an opportunity to establish the combined haematological impact of both symptomatic and asymptomatic parasitaemia since at any point in time the majority of the population can be expected to be aparasitaemic [28]. Moreover, the similar incidence of *P. falciparum* and *P. vivax* infections enables valid comparisons between the two species. In this study, a cross-sectional, community prevalence survey in southern Papua was used to determine adjusted fractions of moderate or severe anaemia (haemoglobin <7 g/dL) attributable to patent *P. falciparum*, *P. vivax* and mixed parasitaemia.

## Methods

### Study site

The geography, climate and demographics of Mimika District and its capital city, Timika, have been described elsewhere [19, 22, 28]. A census in 2004 estimated the local population to be 130,000. Malaria in the region is restricted to lowland areas where it is associated with three mosquito vectors: *Anopheles koliensis, Anopheles farauti* and *Anopheles punctulatus*. The annual incidence of clinical or asymptomatic malaria is approximately 876 episodes per 1000 people, 46 % due to *P. falciparum* and 39 % due to *P. vivax* [28]. Given its equatorial location, the climate in Timika does not change significantly throughout the year and malaria transmission is perennial. At the time of the survey, patients with uncomplicated malaria were generally treated with chloroquine plus sulfadoxine-pyrimethamine, or chloroquine alone. High rates of resistance to these antimalarials are present in both *P. falciparum* and *P. vivax* isolates in the region and therefore both regimens were abandoned in favour of artemisinin-based combination therapy shortly after this survey was conducted. A 14-day course of primaquine was encouraged for those with vivax malaria, however administration was unsupervised.

### Cross-sectional survey methods

Households for this survey were chosen by cluster random sampling. First, the four largest of the 12 sub-districts in Mimika were chosen purposively. Second, the number of clusters required in each sub-district was apportioned according to the relative populations of the sub-districts. In most cases, clusters constituted discrete villages, however, in Mimika Baru the very large population size dictated that villages within this sub-district be sub-divided into census blocks. Once mapped, clusters and 25 houses within each cluster were chosen randomly according to WHO recommendations [29]. Household members were defined as people who lived under one roof, ate from one kitchen and who had resided in the study area for at least 6 months. There were no exclusion criteria. Sociodemographic information, self-reported pregnancy status and history of fever were recorded for all household members using a standardized questionnaire. For household members that were not present at the time of the survey, this information was collected from the head of the household. Those present at the time of the survey each had a single weight measurement using a uniscale. Axillary temperature was recorded using a digital thermometer and a finger-prick sample of blood taken for blood film examination and haemoglobin measurement. Patients with microscopically confirmed malaria were treated according to the Indonesian Ministry of Health Guidelines. Those with anaemia were given iron supplementation according to local protocols. The survey was carried out between July and December 2005.

### Laboratory methods

Blood films were read locally by certified microscopists with at least 10 years experience. A thick smear was considered negative on initial review if no parasites were seen in 100 high power fields. A thin smear was also examined to confirm parasite species and used for quantification if parasitaemia was greater than 200 per 200 WBC. Upon cross-checking 200 high power fields were checked before slides were considered negative. Parasitaemias were calculated assuming a white cell count of 7300 cells/µL. All positive films and 10 % of the negative slides were crosschecked at the National Institute of Health Research and Development reference laboratory in Jakarta. Results that differed were reviewed by the two lead microscopists for final assessment. Haemoglobin concentrations were determined using a calibrated portable Hemacue^®^ machine.

### Statistical analyses

All analyses were done in STATA^®^ version 10.1 (StataCorp, College Station, TX, USA). The primary outcomes in this study were: the absolute haemoglobin concentration, the odds of moderate or severe anaemia and the population-attributable fraction of moderate or severe anaemia associated with infection by the different *Plasmodium* species. For the purposes of this study, a haemoglobin concentration of 7 g/dL was chosen a priori as an appropriate distinction between mild and moderate or severe anaemia (as recommended by Snow and colleagues [7]).

Tests for trend were done using the *ptrend* module for STATA. Univariable linear regression of continuous haemoglobin data was performed for the following exposures: *Plasmodium* species (*P. falciparum, P. vivax* and mixed *P. falciparum/P. vivax* species infections), age group (<1 year, 1 to <5 years, 5 to <15 years, ≥15 years), self-reported ethnicity (non-Papuan, Highland Papuan, Lowland Papuan), pregnancy status, weight for age/gender/ethnicity (≥survey mean, <survey mean) and household income per person (>75th centile, 25th–75th centile, <25th centile). Weight for age/gender/ethnicity was established by creating a nomogram from the survey data.

Multivariable linear and logistic regression analyses of the effect of *Plasmodium* parasitaemia on absolute haemoglobin and odds of moderate or severe anaemia were done for each of the age groups as well as for the study population as a whole. To account for the study design, multivariable models were adjusted for the categorical variable ‘sub-district’ and the variance–covariance matrices of both univariable and multivariable models were adjusted for within-household correlation (giving robust standard errors). Given the effect of menstruation on haemoglobin concentrations, the hypothesis was that gender would modify the relationship between age and anaemia. The interaction between age and a composite variable incorporating gender and pregnancy status was found to be statistically significant and therefore was included in multivariable models of the whole study population.

Adjusted population fractions of moderate-to-severe anaemia attributable to patent parasitaemia were calculated using the aflogit module for STATA [30]. The attributable fractions cannot be summed as the model assumes a mutually exclusive scenario where each risk factor is deemed to be the first to be eliminated [31]. Outputs can therefore be interpreted as the proportion of moderate or severe anaemia that could be prevented by addressing the particular factor of interest in isolation. Patients with *Plasmodium malariae* infections were excluded from all regression models due to small numbers.

## Results

### Parasitaemia

**Fig. 1:**
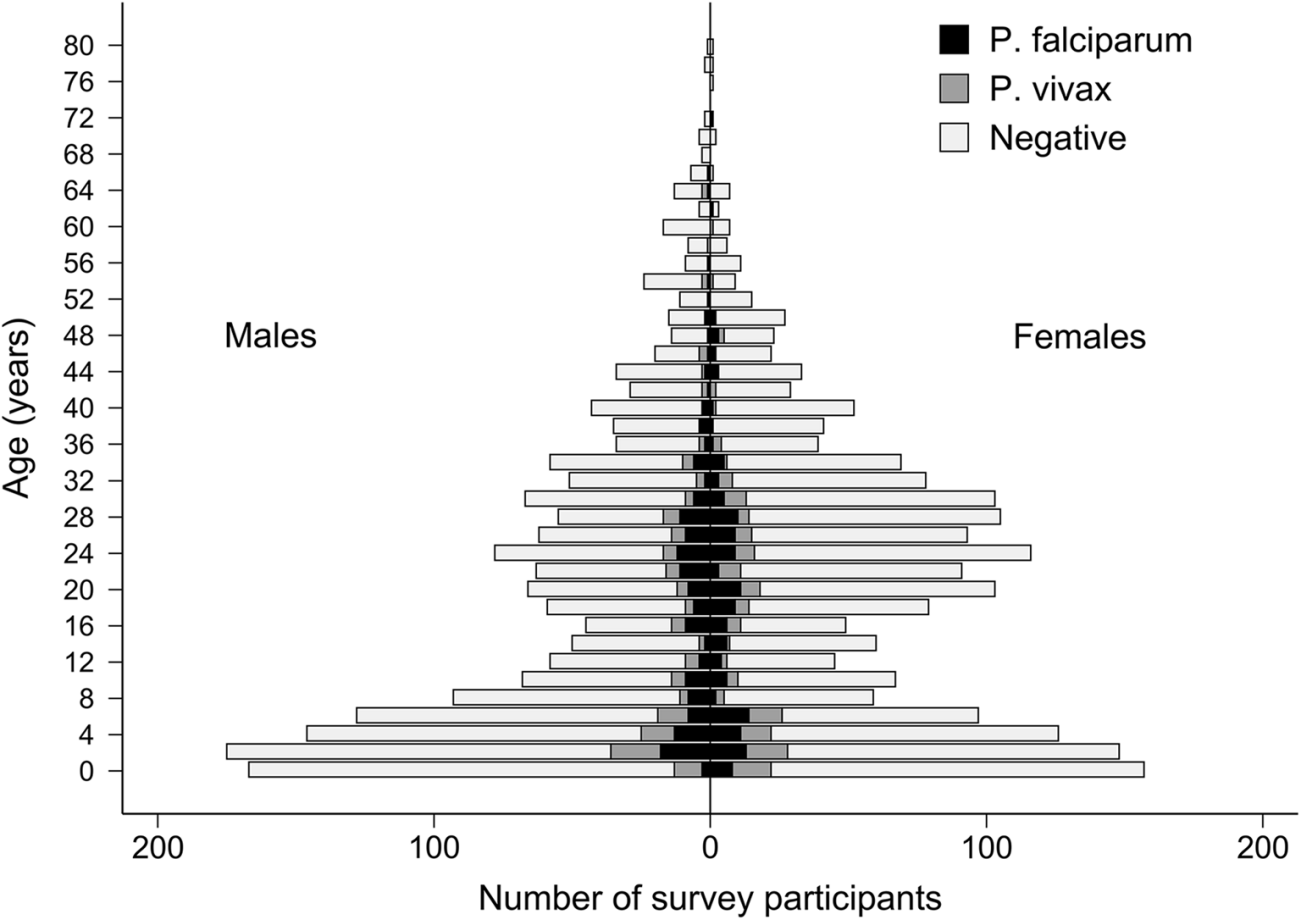
Population structure of those who provided a blood sample by age, gender and presence of parasitaemia

In total, 5255 individuals resided in the 825 households surveyed of whom 3890 (74 %) were present and consented to providing a finger-prick sample of blood (Fig. 1). Those who either declined to provide a sample or were not present at the time of survey were an average of 4 years older (24.7 vs 20.6 years) and more likely to be male (71.2 vs 48.2 %) than their counterparts who provided blood samples. Patent parasitaemia was detected in 17.0 % of the participants, with *P. falciparum* present in 8.1 % (n = 315), *P.* vivax in 6.4 % (n = 250) and mixed infections in 1.9 % (n = 72) (Table 1). A history of fever in the preceding 24 h was present in 33.3 % (105/315) of those with *P. falciparum*, 29.2 % (73/250) with *P. vivax* and 34.7 % (25/72) with mixed infections. More infants (<1 year) and children between the ages of 1 and under 5 years were infected with *P. vivax* compared to *P. falciparum* [12 vs five infants (p = 0.09) and 62 vs 50 children (1 to <5 years (p = 0.3)), respectively] whereas the opposite was observed for all other age groups. After infancy, there was a statistically significant trend to decreasing prevalence of *P.* vivax parasitaemia with increasing age (p = 0.003 for trend) but no such trend for *P. falciparum* (p = 0.13). Unlike in highly endemic regions, there was no age-associated decrease in the likelihood of having concomitant fever with parasitaemia for either species (p = 0.55 for *P. vivax* and p = 0.92 for *P. falciparum*). Overall, a higher proportion of Highland and Lowland Papuans were parasitized (21.2 and 17.3 %, respectively) than non-Papuans (13.1 %; p < 0.001 and p = 0.01, respectively).

**Table 1:** Demographic features of participants in the household survey for whom blood samples were available

N (%)	Gender			Age group (years)				Ethnicity			All
	Male	Female not pregnant	Female pregnant	<1	1–5	5–15	>15	Non-Papuan	Highland Papuan	Lowland Papuan	
Malaria slide negative	1530 (81.6)	1625 (84.2)	74 (85.1)	159 (89.8)	507 (79.0)	668 (81.4)	1895 (84.2)	1582 (86.8)	813 (77.9)	834 (81.5)	3229 (83.0)
*P. falciparum*	166 (8.9)	142 (7.4)	7 (8.0)	5 (2.8)	50 (7.8)	70 (8.5)	190 (8.4)	117 (6.4)	106 (10.2)	92 (9.0)	315 (8.1)
*P. vivax*	122 (6.5)	122 (6.3)	6 (6.9)	12 (6.8)	62 (9.7)	52 (6.3)	124 (5.5)	103 (5.7)	93 (8.9)	54 (5.3)	250 (6.4)
*P. malariae*	13 (0.7)	11 (0.6)	0 (0)	0 (0)	3 (0.5)	11 (1.3)	10 (0.4)	3 (0.2)	9 (0.9)	12 (1.2)	24 (0.6)
Mixed species	43 (2.3)	29 (1.5)	0 (0)	1 (0.6)	20 (3.1)	20 (2.4)	31 (1.4)	18 (1.0)	23 (2.2)	31 (3.0)	72 (1.9)
Total	1874 (48.2)	1929 (95.7)	87 (4.3)	177 (4.6)	642 (16.5)	821 (21.1)	2250 (57.8)	1823 (46.9)	1044 (26.8)	1023 (26.3)	3890 (100)

Totals include the small number of individuals with pure *P. malariae* infections

### Anaemia

**Fig. 2:**
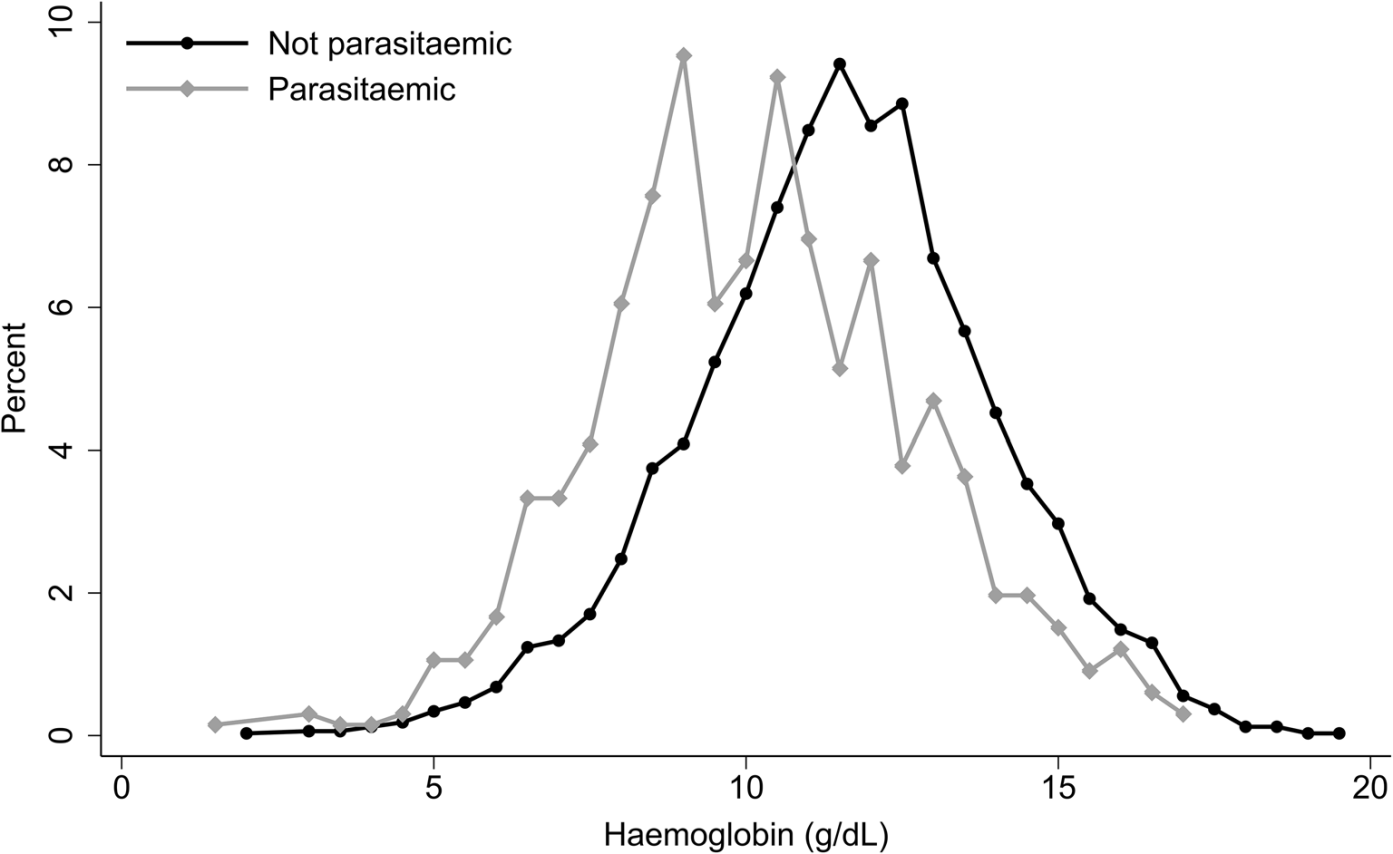
Frequency distribution of haemoglobin concentrations by presence of parasitaemia

The mean haemoglobin in the sample as a whole was 11.0 g/dL (95 % reference range 6.1–15.9 g/dL) (Table 2) with 5.7 % of individuals having a concentration less than 7 g/dL (222/3890). Presence of patent parasitaemia shifted the haemoglobin distribution curve markedly to the left and was associated with a bimodal pattern of haemoglobin concentrations with peaks at 9 and 10.5 g/dL (Fig. 2). Indigenous Papuans and females had significantly lower haemoglobin concentrations than their counterparts in univariable analyses while mean haemoglobin increased with increasing age up to 15 years. No correlation existed between haemoglobin and log_e_ parasite density for *P. falciparum* [Pearson’s correlation coefficient (r) = −0.08, n = 290, 95 % CI −0.19, 0.04, p = 0.2] whereas for *P. vivax* there was a weak negative correlation (r = −0.24, n = 248, 95 % CI −0.35, −0.12, p < 0.001). Those with parasitaemia and fever within the last 24 h (n = 205) had similar mean haemoglobin concentrations as parasitaemic individuals without fever (n = 456) (mean haemoglobin 9.85 vs 9.93 g/dL, p = 0.4). Forty-two per cent (1629/3890) of the survey participants had a history of fever in the last month and these individuals had both a significantly lower mean haemoglobin concentration and an increased unadjusted odds ratio for moderate or severe anaemia compared to those without a history of fever (mean haemoglobin concentration, 10.6 vs 11.3 g/dL, p < 0.001, odds ratio for moderate or severe anaemia, 1.7, 95 % CI 1.3, 2.5, p < 0.001).

**Table 2:** Haemoglobin by presence of parasitaemia (regardless of presence of symptoms) and demographic characteristics

	N	Mean Hb	Std dev	Coef (95 % CI)
*Plasmodium* species				
Negative	3229	11.2	2.41	0
*P. falciparum*	315	9.8	2.43	−1.38 (−1.67, −1.09)
*P. vivax*	250	10.2	2.58	−0.99 (−1.33, −0.65)
*P. malariae*	24	8.9	2.42	–
Mixed infection	72	9.4	2.65	−1.84 (−2.52, −1.17)
Gender and pregnancy status				
Male	1874	11.4	2.70	0.85 (0.69, 1.00)
Female non-pregnant	1929	10.6	2.18	0
Female pregnant	87	10.1	1.86	−0.47 (−0.88, −0.07)
Ethnicity				
Non-Papuan	1823	11.9	2.27	0
Highland Papuan	1044	10.0	2.48	−1.82 (−2.06, −1.58)
Lowland Papuan	1023	10.5	2.32	−1.35 (−1.58, −1.12)
Age (years)				
<1	177	9.4	1.64	−2.35 (−2.63, −2.07)
1–5	642	9.8	1.98	−1.91 (−2.09, −1.72)
5–15	821	10.4	2.03	−1.29 (−1.47, −1.10)
>15	2250	11.7	2.56	0
Weight for age/gender/ethnicity				
≥Mean	1809	11.2	2.47	0
<Mean	2081	10.8	2.47	−0.42 (−0.59, −0.25)
Household income per person				
>75th centile	823	11.3	2.35	0.14 (−0.13, 0.40)
25th–75th centile	1776	11.2	2.51	0
<25th centile	974	10.7	2.43	−0.46 (−0.74, −0.18)
All	3890	11.0	2.48	–

*Hb* haemoglobin, *Std dev* standard deviation, *n* number, *coef* linear regression coefficient, *95* *% CI* 95 % confidence interval

After adjusting for age, gender, ethnicity, pregnancy, and weight for age/gender/ethnicity, presence of *P. falciparum* parasitaemia was associated with an absolute reduction in haemoglobin of 1.16 g/dL (95 % CI 0.91, 1.41, p < 0.001) (Table 3). The corresponding values for *P. vivax* and mixed infections were 0.66 g/dL (95 % CI 0.35, 0.96) and 1.25 g/dL (0.71, 1.80), respectively (p < 0.001 for both). Although numbers were small, *P. vivax* was associated with a large mean reduction in haemoglobin of 1.93 g/dL (95 % CI 0.96, 2.89, p < 0.001) in infants. There was a smaller and non-statistically significant reduction for infants with *P. falciparum* (mean reduction = 0.66 g/dL, 95 % CI −0.86, 2.18, p = 0.4).

**Table 3:** Multiple linear regression showing the effect of *Plasmodium* parasitaemia on mean haemoglobin concentration (g/dL)

	<1 year^a^		1–5 years^a^		5–15 years^a^		>15 years^b^		All^c^	
	Coef (95 % CI)	p	Coef (95 % CI)	p	Coef (95 % CI)	p	Coef (95 % CI)	p	Coef (95 % CI)	p
Malaria										
Negative	0		0		0		0		0	
All species	−1.35 (−2.24, −0.46)	0.003	−1.27 (−1.65, −0.89)	<0.001	−1.08 (−1.45, −0.70)	<0.001	−1.08 (−1.36, −0.80)	<0.001	−0.97 (−1.18, −0.77)	<0.001
*P. falciparum*	−0.66 (−2.18, 0.86)	0.4	−1.60 (−2.07, −1.12)	<0.001	−1.40 (−1.90, −0.89)	<0.001	−1.27 (−1.60, −0.94)	<0.001	−1.16 (−1.41, −0.91)	<0.001
*P. vivax*	−1.93 (−2.89, −0.96)	<0.001	−0.83 (−1.33, −0.33)	0.001	−0.38 (−0.91, 0.14)	0.2	−0.78 (−1.23, −0.32)	0.001	−0.66 (−0.96, −0.35	<0.001
Mixed	1.60 (1.01,2.19)	<0.001	−1.87 (−2.88, −0.85)	<0.001	−1.82 (−2.39, −1.26)	<0.001	−1.14 (−2.04, −0.25)	0.01	−1.25 (−1.80, −0.71)	<0.001

*Coef* coefficient, *95* *% CI* 95 % confidence interval

^a^Models also include gender, ethnicity (non-Papuan, Highland Papuan, Lowland Papuan) and weight for age/gender/ethnicity (<mean, ≥mean)

^b^Models also include gender, ethnicity (non-Papuan, Highland Papuan, Lowland Papuan) and weight for age/gender/ethnicity (<mean, ≥mean) and pregnancy status

^c^Model also includes age (as a continuous variable) by gender/pregnancy status, ethnicity (non-Papuan, Highland Papuan, Lowland Papuan) and weight for age/gender/ethnicity (<mean, ≥mean)

Table 4 shows adjusted odds ratios for having haemoglobin less than 7 g/dL. Individuals with mixed infections were most likely to have moderate or severe anaemia [Adjusted Odds Ratio (AOR) = 3.18, 95 % CI 1.67, 6.07, p < 0.001] followed by patients with *P. falciparum* (AOR = 2.29, 95 % CI 1.52, 3.44, p < 0.001) and *P. vivax*, respectively (AOR = 1.93, 95 % CI 1.14, 3.25, p = 0.01). For infants with *P. vivax* the AOR was 47.7 (95 % CI 3.19, 712, p = 0.005) and for infants with *P. falciparum* it was 6.02 (95 % CI 0.54, 67.0, p = 0.1).

**Table 4:** Adjusted odds ratios for having a haemoglobin concentration less than 7 g/dL

	<1 year^a^		1–5 years^a^		5–15 years^a^		>15 years^b^		All^c^	
	AOR (95 % CI)	p	AOR (95 % CI)	p	AOR (95 % CI)	p	AOR (95 % CI)	p	AOR (95 % CI)	p
Parasite negative	1		1		1		1		1	
Any species	12.7 (2.36, 68.8)	0.003	2.26 (1.12, 4.59)	0.02	2.47 (1.22, 5.01)	0.01	2.01 (1.20, 3.35)	0.008	2.25 (1.61, 3.15)	<0.001
*P. falciparum*	6.02 (0.54, 67.0)	0.1	2.64 (1.11, 6.26)	0.03	3.02 (1.23, 7.42)	0.02	1.08 (0.95, 3.44)	0.07	2.29 (1.52, 3.44)	<0.001
*P. vivax*	47.7 (3.19, 712)	0.005	1.37 (0.48, 3.77)	0.5	1.84 (0.62, 5.49)	0.3	2.12 (0.93, 4.82)	0.07	1.93 (1.14, 3.25)	0.01
Mixed species	–		4.46 (1.49, 13.3)	0.008	2.11 (0.46, 9.62)	0.3	2.87 (0.64, 12.9)	0.2	3.18 (1.67, 6.07)	<0.001

*AOR* adjusted odds ratio, *95* *% CI* 95 % confidence interval

^a^Models also include gender, ethnicity (non-Papuan, Highland Papuan, Lowland Papuan) and weight for age/gender/ethnicity (<mean, ≥mean)

^b^Models also include gender, ethnicity (non-Papuan, Highland Papuan, Lowland Papuan) and weight for age/gender/ethnicity (<mean, ≥mean) and pregnancy status

^c^Model also includes age (as a continuous variable) by gender/pregnancy status, ethnicity (non-Papuan, Highland Papuan, Lowland Papuan) and weight for age/gender/ethnicity (<mean, ≥mean)

### Population-attributable fractions of anaemia due to malaria

**Fig. 3:**
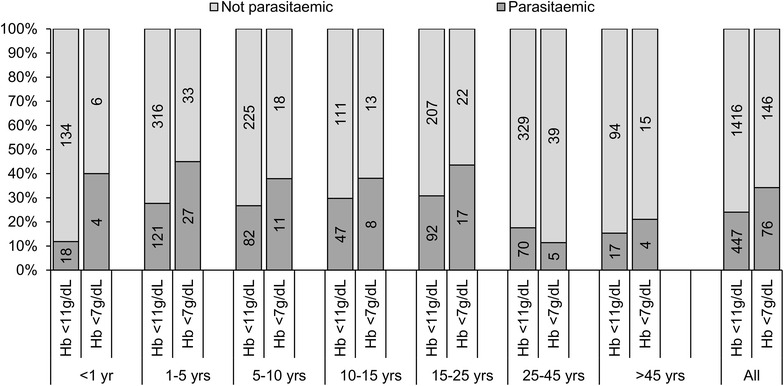
Proportion of participants with haemoglobin concentrations <11 and <7 g/dL who had parasitaemia (*bar labels* = absolute numbers)

Patent parasitaemia due to any species of *Plasmodium* was responsible for 16.7 % (95 % CI 8.52, 24.2 %) of cases of moderate or severe anaemia in this study (Table 5). The corresponding values for *P. falciparum, P. vivax* and mixed infections were 8.35 % (95 % CI 3.25, 13.2 %), 4.94 % (95 % CI 0.18, 9.48 %) and 3.41 % (95 % CI 0.08, 5.95 %), respectively. The attributable fraction was greatest in infants (34.4 %, 95 % CI −3.30, 58.3 %) and decreased with increasing age thenceforth. *Plasmodium vivax* was responsible for greater than three times the proportion of moderate or severe anaemia in infants compared with *P. falciparum* [27.6 % (95 % CI −3.20, 49.2 %) versus 7.94 % (−5.87, 20.0 %)] although the precision of these estimates was poor. Figure 3 indicates that in general, a greater proportion of moderate or severe anaemia (haemoglobin <7 g/dL) is attributable to malaria than mild anaemia (haemoglobin <11 g/dL).

**Table 5:** Adjusted population-attributable fractions of moderate or severe anaemia (haemoglobin concentration less than 7 g/dL) by presence or absence of *Plasmodium* parasitaemia

	<1 year^a^		1–5 years^a^		5–15 years^a^		>15 years^b^		All^c^	
	aPAF	95 % CI	aPAF	95 % CI	aPAF	95 % CI	aPAF	95 % CI	aPAF	95 % CI
Any species	34.4	−3.30, 58.3	23.2	3.34, 39.0	19.4	0.99, 34.5	12.1	1.80, 21.4	16.7	8.52, 24.2
*P. falciparum*	7.94	−5.87, 20.0	10.5	−0.66, 20.5	13.7	0.04, 25.5	5.34	−1.69, 11.9	8.35	3.25, 13.2
*P. vivax*	27.6	−3.20, 49.2	4.07	−6.21, 13.4	4.28	−4.90, 12.7	4.16	−2.14, 10.1	4.94	0.18, 9.48
Mixed species	–		8.55	1.93, 14.7	1.99	−3.08, 6.82	1.80	−1.91, 5.37	3.41	0.08, 5.95

*aPAF* adjusted population-attributable fraction, *95* *% CI* 95 % confidence interval

^a^Models also include gender, ethnicity (non-Papuan, Highland Papuan, Lowland Papuan) and weight for age/gender/ethnicity (<mean, ≥mean)

^b^Models also include gender, ethnicity (non-Papuan, Highland Papuan, Lowland Papuan) and weight for age/gender/ethnicity (<mean, ≥mean) and pregnancy status

^c^Model also includes age (as a continuous variable) by gender/pregnancy status, ethnicity (non-Papuan, Highland Papuan, Lowland Papuan) and weight for age/gender/ethnicity (<mean, ≥mean)

## Discussion

Despite comparatively low-level endemicity, patent parasitaemia in southern Papua is associated with 17 % of all haemoglobin concentrations under 7 g/dL. In infants and young children, the corresponding proportions rise to 34 and 23 %, respectively. Although *P. vivax* is less prevalent than *P. falciparum* overall, this study has shown that in Mimika District it is the commoner species in children under 5 years of age and that it is associated with a higher population-attributable fraction of anaemia in infants. This study also suggests that mixed species infections are associated with a greater reduction in haemoglobin than *P. falciparum* or *P. vivax* infections alone.

In this survey, 17 % of individuals were parasitaemic and approximately one-third of these were febrile. Presence of fever did not confer a greater risk of anaemia. Although parasitaemia was strongly associated with anaemia, the population attributable fractions presented are still likely to represent under-estimates of the true total effect of malaria on haemoglobin concentrations in the community. Full haematological recovery takes several weeks following acute malaria [32] suggesting that many aparasitaemic individuals may have been experiencing the haematological after-effects of recent malaria infection. Nearly a half of the survey participants had a history of fever in the preceding month and these individuals had significantly lower haemoglobin concentrations than those without a history of fever. Since 35 % of fevers in the community are estimated to be due to malaria, parasitaemia is likely to have been responsible for a sizeable, but unmeasured, proportion of all reduced haemoglobin concentrations in the aparasitaemic group.

The estimated adjusted population-attributable fractions of moderate or severe anaemia in infants were based on small numbers. Nevertheless, the finding that *P. vivax* accounts for a greater fraction of anaemia than *P. falciparum* is in agreement with results from the local hospital [1, 19, 33] as well as a previous cross-sectional survey in Papua New Guinea [34]. At the hospital, *P. vivax* is the most common cause of malaria-related admission in the first year of life and produces an equal or greater reduction in haemoglobin than *P. falciparum* [1, 19, 33]. Others have also observed that morbidity from vivax malaria is maximal at a much younger age than falciparum malaria [35–38], a phenomenon that Maitland and colleagues speculate is due to greater ease of transmission and more rapid acquisition of immunity [39]. In keeping with this hypothesis, it is the authors’ view that both the greater prevalence of vivax malaria and the severity of the associated anaemia in infancy observed in this study are not chance findings but are related to multiple relapses causing repetitive insults to the haematological system and inducing early development of immunity. Two sources of evidence from this study support this hypothesis. First, there was a statistically significant reduction in the prevalence of *P. vivax* parasitaemia with age, whereas there was no such reduction for *P. falciparum*. Second, infants with *P. vivax* parasitaemia in this study had had significantly more episodes of fever in the last month than infants with *P. falciparum* (median 1 vs 0 episodes, p = 0.007). The subsequent decline in the fraction of anaemia attributable to either species of *Plasmodium* with increasing age is likely to relate to three main factors: the acquisition of some degree of immunity, especially in the case of vivax malaria, the increasing importance of alternative causes of anaemia, such as intestinal helminthiasis and chronic infections, and lastly the increasing likelihood that lack of parasitaemia represents a state of remission or a period between primary infections rather than a state of complete malaria naivety.

This study showed that mixed species infections were associated with a greater drop in haemoglobin and a higher risk of moderate or severe anaemia than infections with *P. falciparum* or *P. vivax* alone. This finding is consistent with other work from Papua [1, 19] and Papua New Guinea [40] but in direct contradiction to research carried out in Thailand and elsewhere [39]. Concomitant infection with *P. vivax* in northern Thailand has been postulated to attenuate the risk of severe anaemia secondary to *P. falciparum* infection, possibly due to some degree of cross-species immunity [32, 39]. In Papua, where endemicity of both species is higher, mixed infection may reflect a greater likelihood of having had multiple recent malaria infections (likely due to *P. vivax*), driving deeper levels of anaemia.

This study has several limitations. Due to the cross-sectional design it was not possible to draw solid conclusions about the direction of the observed associations. Although anaemia is an established sequel of both falciparum and vivax malaria, there is evidence that iron deficiency anaemia reduces the risk of falciparum malaria [41] and conversely, that administering iron supplements to iron-replete individuals may slightly increase the risk [42, 43]. The comparative effect of this reverse causation is likely to be small since there were no special community-wide supplementation programmes at the time of the survey.

The population-attributable fractions were estimated using odds ratios as approximations of relative risk. Since moderate or severe anaemia was not a particularly rare outcome, this may have resulted in slight overestimation of the attributable fractions, particularly for 1–5 years olds who had a prevalence of moderate or severe anaemia of 9.4 %.

Selection bias may have affected the population-attributable fractions due to their heavy reliance on the prevalence of parasitaemia in the sample. Those who did not provide a blood sample (mostly due to absence at the time of the survey) tended to be older males. Overall, there was relatively little effect of increasing age or gender on the odds of parasitaemia, however those who did not provide a blood sample could conceivably have been at greater risk of malaria acquisition due to behavioural or lifestyle factors. If this were true, the fraction of anaemia attributable to malaria may have been underestimated.

Several potentially important confounders could not be controlled for in analyses. Infestation with intestinal helminths has been shown to cause an additive reduction in haemoglobin concentrations in malaria co-infected children [44]. Although there is a great deal of geospatial overlap between malaria and intestinal helminths [45], the immunological relationship remains less clear [46, 47]. Two studies by Nacher and Spiegel, respectively, suggest that the presence of intestinal helminths increases the risk of falciparum malaria by a factor of between 1.5 and 2.2 [48, 49]. Even if this is the case, the results of this study for infants and children under 5 years are unlikely to be significantly confounded since intestinal helminth density does not typically peak until early adulthood and a previous study showed minimal impact of intestinal helminthiasis on haemoglobin concentrations before 30 months of age [50].

Haemoglobin and red cell abnormalities as a whole are protective against severe malarial anaemia [51–53] but their effect on the risk of uncomplicated *Plasmodium* infection is less clear and may differ between species [51, 54, 55]. Since these disorders are themselves risk factors for anaemia, differences in their distribution between participants with and without parasitaemia could potentially have confounded the models.

Incorporating weight for age/gender/ethnicity into the multivariable regression models should have accounted for at least some of the potential confounding caused by iron deficiency anaemia. Since iron deficiency is thought to be protective against *Plasmodium* infection, any residual confounding is likely to have biased the results towards the null. Finally, the effects of chronic disease and bacteraemia could not be controlled for in this analysis. The former is unlikely to have been important in the younger age groups and bacteraemia would be expected to be rare in the community setting.

## Conclusions

Despite comparatively low-level *Plasmodium* endemicity, patent parasitaemia (whether symptomatic or not) is an important and preventable cause of anaemia in southern Papua. Young children bear the brunt of this burden but the haematological effects also extend into adulthood. *Plasmodium vivax* is an especially important cause of anaemia in infants, probably because it causes recurrent disease prior to the onset of immunity. Since infancy is a time of increased susceptibility to infectious diseases as well as rapid physical and neurological development, anaemia associated with vivax malaria may be an important and under-estimated contributor to indirect malaria mortality and developmental morbidity in regions where this species is prevalent.

## Abbreviations

AOR: adjusted odds ratio
CI: confidence interval
OR: odds ratio
R: Pearson’s correlation coefficient
